# Revealing universal quantum contextuality through communication games

**DOI:** 10.1038/s41598-019-53701-5

**Published:** 2019-11-26

**Authors:** A. K. Pan

**Affiliations:** National Institute of Technology Patna, Ashok Rajhpath, Patna, 800005 India

**Keywords:** Quantum mechanics, Qubits

## Abstract

An ontological model of an operational theory is considered to be universally noncontextual if both preparation and measurement noncontextuality assumptions are satisfied in that model. In this report, we first generalize the logical proofs of quantum preparation and measurement contextuality for qubit system for any odd number of preparations and measurements. Based on the logical proof, we derive testable universally non-contextual inequalities violated by quantum theory. We then propose a class of two-party communication games and show that the average success probability of winning such games is solely linked to suitable Bell expression whose local bound is greater than universal non-contextual bound. Thus, for a given state, even if quantum theory does not exhibit non-locality, it may still reveal non-classicality by violating the universal non-contextual bound. Further, we consider a different communication game to demonstrate that for a given choices of observables in quantum theory, even if there is no logical proof of preparation and measurement contextuality exist, the universal quantum contextuality can be revealed through that communication game. Such a game thus test a weaker form of universal non-contextuality with minimal assumption.

## Introduction

Two famous no-go theorems play an important role in quantum foundations as they demonstrate how quantum world differs from the classical ones by ruling out the existence of particular kinds of physical models. Bell’s theorem^1^ is the first that provides a conflict between the quantum theory and a notion of classicality widely known as local realism. The Kochen and Specker (KS) theorem^2,3^ proves an inconsistency between the quantum theory and another notion of classicality known as non-contextual realism. Quantum non-locality found its application in device-independent cryptography^4–6^, certification of randomness^7–9^, self-testing^10,11^, certification of dimension of Hilbert space^12^. On the other hand, quantum contextuality also provides advantage in communication games^13–15^ and in quantum computation^16,17^. While the demonstration of Bell’s theorem requires two or more space-like separated systems, the KS theorem can also be proven for a single system having dimension of the Hilbert space *d* ≥ 3. The original KS proof was demonstrated by using 117 projectors for qutrit system. Later, simpler versions and varients of it using lower number of projectors have been provided^18–24^.

The traditional KS proof of contextulity has a limited scope of applicability due to the following reasons. First, along with the assumption of measurement non-contextuality it additionally requires outcome determinism for sharp measurement in an ontological model. Second, it is not the model of arbitrary operational theory, rather specific to quantum theory. Third, it is not applicable to the generalized measurements, i.e., for POVMs. The traditional notion of KS non-contextuality was generalized by Spekkens^25^ for any arbitrary operational theory and extended the formulation to the transformation and preparation non-contextuality.

The ontological model of an operational theory was coherently formulated by Harrigan and Spekkens^26^. Let there is a set of preparation procedures ${\mathscr{P}}$, a set of measurement procedures ${\mathscr M}$ and a set of outcomes ${\mathscr{K}}$_M_. Given a preparation procedure $P\in {\mathscr{P}}$ and a measurement procedures *M* ∈ ${\mathscr M}$, an operational theory assigns probability *p*(*k*|*P*, *M*) of occurrence of a particular outcome *k* ∈ $\mathscr{K}$_M_. For example, in quantum theory, a preparation procedure produces a density matrix *ρ* and measurement procedure is in general described by a suitable POVM *E*_*k*_. The probability of occurrence of a particular outcome *k* is determined by the Born rule, i.e., *p*(*k*|*P*, *M*) = *Tr*[*ρE*_*k*_]. In this paper we restrict our discussion in a particular operational theory, i.e., in quantum theory.

In an ontological model of quantum theory, it is assumed that whenever *ρ* is prepared by a preparation procedure $P\in {\mathscr{P}}$ a probability distribution *μ*_*P*_(*λ*|*ρ*) in the ontic space is prepared, satisfying ${\int }_{\Lambda }{\mu }_{P}(\lambda |\rho )d\lambda =1$ where *λ* ∈ Λ and Λ is the ontic state space. The outcome *k* is distributed as a response function *ξ*_*M*_(*k*|*λ*, *E*_*k*_) satisfying ${\sum }_{k}\,{\xi }_{M}(k|\lambda ,{E}_{k})=1$ where a POVM *E*_*k*_ is realized through a measurement procedure *M* ∈ ${\mathscr M}$. A viable ontological model should reproduce the Born rule, i.e., ∀*ρ*, ∀*E*_*k*_ and ∀*k*, ${\int }_{\Lambda }\,{\mu }_{P}(\lambda |\rho ){\xi }_{M}(k|\lambda ,{E}_{k})d\lambda =Tr[\rho {E}_{k}]$.

An ontological model can be assumed to be non-contextual as follows^25^. If two experimental procedures are operationally equivalent, they have equivalent representations in the ontological model. An ontological model of quantum theory is assumed to be measurement non-contextual if ∀*Pp*(*k*|*P*, *M*) = *p*(*k*|*P*, *M*′) ⇒ ∀*P ξ*_*M*_(*k*|*λ*, *E*_*k*_) = *ξ*_*M*′_(*k*|*λ*, *E*_*k*_), where *M* and *M*′ are two measurement procedures realizing the same POVM *E*_*k*_. KS non-contextuality assumes the aforementioned measurement non-contextuality for the sharp measurements along with the deterministic response functions for projectors. Similarly, an ontological model of quantum theory can be considered to be preparation non-contextual if ∀*M*:*p*(*k*|*P*, *M*) = *p*(*k*|*P*′, *M*) ⇒ ∀*M μ*_*P*_(*λ*|*ρ*) = *μ*_*P*′_(*λ*|*ρ*), where *P* and *P*′ are two distinct preparation procedures preparing same density matrix *ρ*. In an ontological model of quantum theory, the preparation non-contextuality implies the outcome determinism for sharp measurements^25^. Also, any KS proof can be considered as a proof of preparation contextuality but converse does not hold. In this sense, preparation non-contextuality is a stronger notion than traditional KS non-contextuality^27^. Very recently, it is also shown^28^ that any ontological model satisfying both the assumptions of preparation and measurement non-contextuality cannot reproduce all quantum statistics, even if the assumption of outcome determinism for sharp measurement is dropped^28^. Experimental test of such an universal non-contextuality has also been provided which are free from idealized assumptions of noiseless measurements and exact operational equivalences^29^.

In this work, we first generalize the universal contextuality proof demonstrated in^29^ for any arbitrary odd number of preparations and measurements in a qubit system. We then propose a class of communication games played between two spatially separated parties Alice and Bob and show that the average success probability of winning the game is solely dependent on a Bell expression. The local bound of such a Bell expression gets reduced if universal non-contextuality is assumed. Thus, for a given state, even if quantum theory does not violate local realist bound but it may still reveal non-classicality by violating the universal non-contextuality. We further point out that for a given choices of projectors corresponding to a suitable set of dichotomic observables, if there exists a logical proof of preparation contextuality for mixed state then there will be an inherent interplay between the preparation contextuality for mixed and pure states. In other words, assumption of preparation non-contextuality for mixed state in the logical proof automatically assumes preparation contextuality for pure states.A true test of universal contextuality should be free from such inconsistency. Further, we demonstrate that for a suitable choices of states and observables when there is no logical proof of preparation and measurement contextuality exist, universal quantum contextuality c an still be revealed through a suitable communication game.

We first encapsulate the notion of preparation and measurement non-contextuality in an ontological model of quantum theory which was first put forwarded by Spekkens^25^.

## Logical Proofs of Measurement and Preparation Contextuality

As already mentioned that the KS proof is restricted to deterministic ontological models and does not work for generalized measurement. In other words, KS proof is valid for commuting contexts and thus the Hilbert space dimension needs to be more than two^1,3^. It is pointed out in^25^ that the natural question should be whether in a non-deterministic ontological model the probabilities of different outcomes for a given *λ* depend on the compatible context instead of commuting context. Assuming the indeterministic response functions for POVMs (rigoursly justified in^30^) measurement contextuality can be demonstrated even for two-dimensional Hilbert space.

Let *M*_*1*_, *M*_*2*_, *M*_*3*_∈ ${\mathscr M}$ are three measurement procedures in quantum theory realizing three non-degenerate dichotomic observables *A*_1_, *A*_2_, *A*_3_ respectively. The projectors corresponding to *A*_*t*_ are ${P}_{{A}_{t}}^{\alpha }=\frac{{\mathbb{I}}+\alpha {A}_{t}}{2}$ where *t* = 1, 2, 3 and *α* ∈ {+, −} satisfying

1 $${\mathbb{I}}={P}_{{A}_{t}}^{+}+{P}_{{A}_{t}}^{-};\,{\rm{with}}\,{P}_{{A}_{t}}^{+}{P}_{{A}_{t}}^{-}=0.$$

Since $Tr[{\mathbb{I}}\rho ]=1$ for any arbitrary *ρ*, then in an ontological model $\xi (\alpha |{\mathbb{I}},\lambda )=1$ for every ontic state *λ* ∈ Λ. Thus, the response functions follow the equivalent relations

2 $$1={\xi }_{{M}_{t}}(\,+\,|{P}_{{A}_{t}}^{+},\lambda )+{\xi }_{{M}_{t}}(\,-\,|{P}_{{A}_{t}}^{-},\lambda )\,{\rm{with}}\,{\xi }_{{M}_{t}}(\,+\,|{P}_{{A}_{t}}^{+},\lambda ){\xi }_{{M}_{t}}(\,-\,|{P}_{{A}_{t}}^{-},\lambda )=0.$$

Now, consider another measurement procedure M_*_, satisfying

3 $$\{\frac{{\mathbb{I}}}{2},\frac{{\mathbb{I}}}{2}\}=\{\frac{1}{3}\mathop{\sum }\limits_{t\mathrm{=1}}^{3}\,{P}_{{A}_{t}}^{+},\,\frac{1}{3}\mathop{\sum }\limits_{t\mathrm{=1}}^{3}\,{P}_{{A}_{t}}^{-}\}$$

where $\frac{{\mathbb{I}}}{2}$ is a POVM. Note that, this is only possible if ${\sum }_{t\mathrm{=1}}^{3}\,{A}_{t}=0$. A simple choice of qubit observables along trine spin axes satisfies this requirement. The corresponding response function for POVMs follow measurement non-contextuality, so that

4 $$\{\frac{1}{2},\frac{1}{2}\}=\{\frac{1}{3}\mathop{\sum }\limits_{t\mathrm{=1}}^{3}\,{\xi }_{{M}_{\ast }}(\,+\,|{P}_{{A}_{t}}^{+},\lambda ),\,\frac{1}{3}\mathop{\sum }\limits_{t\mathrm{=1}}^{3}\,{\xi }_{{M}_{\ast }}(\,-\,|{P}_{{A}_{t}}^{-},\lambda )\}.$$

If the model is outcome deterministic for sharp measurement then each *ξ*(*α*|*P*_*At*_^*α*^, *λ*) ∈ {0, 1} and if measurement non-contextual then *ξ*(*α*|*P*_*At*_^*α*^, *λ*) in Eq. (2) remains same as in Eq. (4). Importantly, no deterministic assignment can satisfy Eq. (4) and thus needs to be dropped. However, allowing *ξ*(*λ*|*P*_*At*_^*α*^) ∈ [0, 1] measurement non-contextuality is satisfied.

It is important to note that the KS proof does not make any reference to the notion of preparation non-contextuality^25^ which should be the starting point of any ontological model to ensure that the *λ* distribution for operationally equivalent preparations are the same to make the measurement non-contextuality assumption justified for any *λ*.

Let three preparation procedures ${P}_{1},{P}_{2},{P}_{3}\in {\mathscr{P}}$ produce six pure qubit states {*ρ*_*At*_^*α*^} satisfying following three relations

5 $$\frac{{\mathbb{I}}}{2}=\frac{1}{2}({\rho }_{{A}_{t}}^{+}+{\rho }_{{A}_{t}}^{-}).$$

In an ontological model, using convexity property one can write

6 $${\mu }_{{P}_{t}}(\lambda |\frac{{\mathbb{I}}}{2})=\frac{1}{2}({\mu }_{{P}_{t}}(\lambda |{\rho }_{{A}_{t}}^{+})+{\mu }_{{P}_{t}}(\lambda |{\rho }_{{A}_{t}}^{-}))$$

which we call trivial preparation non-contextuallity condition.

The maximally mixed state $\frac{{\mathbb{I}}}{2}$ can also be prepared by two more preparation procedures *P*_4_ and *P*_5_ are of the following form

7 $$\frac{{\mathbb{I}}}{2}=\frac{1}{3}\mathop{\sum }\limits_{t=1}^{3}\,{\rho }_{{A}_{t}}^{+};\,\frac{{\mathbb{I}}}{2}=\frac{1}{3}\mathop{\sum }\limits_{t=1}^{3}\,{\rho }_{{A}_{t}}^{-}.$$

Using the convexity property of the *λ* distributions

8 $${\mu }_{{P}_{4}}(\lambda |\frac{{\mathbb{I}}}{2})=\frac{1}{3}\mathop{\sum }\limits_{t=1}^{3}\,{\mu }_{{P}_{4}}(\lambda |{\rho }_{{A}_{t}}^{+});\,{\mu }_{{P}_{5}}(\lambda |\frac{{\mathbb{I}}}{2})=\frac{1}{3}\mathop{\sum }\limits_{t=1}^{3}\,{\mu }_{{P}_{5}}(\lambda |{\rho }_{{A}_{t}}^{-})$$

which we call non-trivial preparation noncontextuality conditions. Without such non-trivial conditions logical proof of preparation contextuality for mixed state cannot be revealed in this example.

It is indistinguishable in quantum theory by any measurement which of the five preparation procedures is used to prepare the mixed state $${\mathbb{I}}/2$$. Equivalently, in a preparation non-contextual model, it can be assumed that *λ* distributions corresponding to the five preparation procedures are the same, i.e., $${\mu }_{{P}_{1}}(\lambda |\frac{{\mathbb{I}}}{2})={\mu }_{{P}_{2}}(\lambda |\frac{{\mathbb{I}}}{2})={\mu }_{{P}_{3}}(\lambda |\frac{{\mathbb{I}}}{2})=$$$${\mu }_{{P}_{4}}(\lambda |\frac{{\mathbb{I}}}{2})={\mu }_{{P}_{5}}(\lambda |\frac{{\mathbb{I}}}{2})\equiv \nu (\lambda |\frac{{\mathbb{I}}}{2})$$. We make it specific here by denoting the above assumption as mixed-state preparation non-contextuality. Importantly, for a logical proof of preparation non-contextuality for mixed state, one requires to assume preparation non-contextuality of *λ* distribution *μ*(*λ*|{*ρ*_*At*_^*α*^}) corresponding to the pure states^25^. Assume that there exist a *λ* for which $$\nu (\lambda |\frac{{\mathbb{I}}}{2})\, > 0$$ and for the same *λ* assume *μ*(*λ*|{*ρ*_*At*_^+^}) > 0. Then by using *μ*(*λ*|*ρ*_*At*_^+^)*μ*(*λ*|*ρ*_*At*_^−^) = 0, from Eq. (8) one finds $${\mu }_{{P}_{5}}(\lambda |\frac{{\mathbb{I}}}{2})=0$$ which is in contradiction with the above assignment. Similar contradiction can be found for any assignment of positive probability distribution for any *λ*. However, if preparation contextuality for pure states is assumed, i.e., given a *λ* if *μ*(*λ*|{*ρ*_*At*_^*α*^}) change their support in *P*_*t*_ and *P*_5_ or *P*_4_, the preparation non-contextuality for mixed state will be satisfied in an ontological model.

There is a fundamental difference between the logical proofs of measurement and preparation contextuality. Measurement non-contextuality for projector and POVMs may be satisfied in an ontological model if determinism is sacrificed. We note here that in Cabello’s^18^ elegant proof using 18 vector can also be shown measurement non-contextual if all the response functions are taken to be 1/4. On the other hand, in logical proof of preparation contextuality the assumption of preparation non-contextuality for pure states dictates preparation contextuality for mixed states and vice versa. Hence, such a contradiction is within the ontological model without recourse to any operational theory. Thus, if one wishes to propose a true test of the reproducibility of quantum theory by a universal non-contextual model, the assumptions of outcome determinism and inherent contradiction between preparation non-contextuality for pure and mixed states should be avoided. Such an attempt was made in^28,29^ through a testable inequality which is verified experimentally^29^.

## Universal Non-Contextual Inequality in (3, 3) Scenario

We now recapitulate the non-contextual inequality in three-preparation and three- measurement scenario (henceforth, (3, 3) scenario)^29^. We start from a simple observation. Let a preparation procedure preparing the maximally mixed state ${\mathbb{I}}\mathrm{/2}$ and a measurement of a qubit observable, so that, ${\mathbb{I}}={P}_{{A}_{t}}^{+}+{P}_{{A}_{t}}^{-}$. Since $Tr[\frac{{\mathbb{I}}}{2}\mathrm{.}{\mathbb{I}}]=1$ one can write $\frac{1}{2}Tr[({\rho }_{{A}_{t}}^{+}+{\rho }_{{A}_{t}}^{-})\cdot ({P}_{{A}_{t}}^{+}+{P}_{{A}_{t}}^{-})]=1$ irrespective of *P* and *M*. Here *P*_*At*_^*α*^ is equal to *ρ*_*At*_^*α*^ but to denote preparation we use *ρ*_*At*_^*α*^. In (3, 3) scenario, the average correlation can be written as

9 $${({\Delta }_{3})}_{Q}=\frac{1}{6}\mathop{\sum }\limits_{t\mathrm{=1}}^{3}\,Tr({\rho }_{{A}_{t}}^{+}{P}_{{A}_{t}}^{+}+{\rho }_{{A}_{t}}^{-}{P}_{{A}_{t}}^{-}).$$

Due to the perfect correlation in quantum theory each *Tr*[*ρ*_*At*_^*α*^*P*_*At*_^*α*^] = 1, one has (Δ_3_)_*Q*_ = 1. In an ontological model

10 $${\Delta }_{3}=\frac{1}{6}\mathop{\sum }\limits_{t\mathrm{=1}}^{3}\,\sum _{\alpha \in \{+,-\}}\,\sum _{\lambda \in \varLambda }\,{\xi }_{{M}_{t}}(\alpha |{P}_{{A}_{t}}^{\alpha },\lambda ){\mu }_{{P}_{t}}(\lambda |{\rho }_{{A}_{t}}^{\alpha }).$$

To get perfect correlation, each *ξ*_*Mt*_(*α*|*P*_*At*_^*α*^, *λ*) should produce deterministic outcome. By assuming mixed state preparation non-contextuality $\nu (\lambda |{\mathbb{I}}/\mathrm{2)}=\frac{1}{2}(\mu (\lambda |{\rho }_{{A}_{t}}^{+})+\mu (\lambda |{\rho }_{{A}_{t}}^{-}))$ independent of *t* and by noting that there is an upper bound on each *ξ*_*Mt*_(*α*|*P*_*At*_^*α*^, *λ*) independent of the outcome *α*, one has *ξ*_*Mt*_(*α*|*P*_*At*_^*α*^, *λ*) ≤ *η*(*P*_*At*_, *λ*). Equation (10) can then be written as

11 $${({\Delta }_{3})}_{unc}\le \mathop{\lambda \in \Lambda }\limits^{max}(\frac{1}{3}\sum _{t\in \mathrm{\{1,2,3\}}}\,\eta ({P}_{{A}_{t}},\lambda )).$$

If the response functions are constrained by Eqs. (2) and (4), we have (*η*(*P*_*A*1_, *λ*), *η*(*P*_*A*2_, *λ*), *η*(*P*_*A*3_, *λ*)) = (1, 1/2, 1). Such an indetrministic assignment in an universal non-contextual model provide (Δ_3_)_*unc*_ ≤ 5/6. Thus, an universal non-contextual model cannot reproduce quantum statistics. This result is verified in a recent experiment^29^.

We shall shortly generalize the above proof for a qubit system for any arbitrary odd number of observables. Before that, we provide a scheme to reveal quantum universal contextuality by using a two-party communication game as a tool.

## Results

### A communication game in (3, 3) scenario

Let Alice and Bob are two parties having input *x* ∈ {1, 2, 3} and *y* ∈ {1, 2, 3} respectively and their respective outputs are *a* ∈ {−1, 1} and *b* ∈ {−1, 1}. The wining rule is the following; if *x* = *y* the outputs satisfies *a* ≠ *b* and if *x* ≠ *y* the outputs satisfies *a* = *b*. Let Alice and Bob share a maximally entangled state and the input *x* ∈ {1, 2, 3} corresponds to the observables *A*_1_, *A*_2_, *A*_3_ and similarly *y* ∈ {1, 2, 3} corresponds to *B*_1_, *B*_2_, *B*_3_. Alice measures one of *A*_1_, *A*_2_, *A*_3_ to produce six pure qubit states {*ρ*_*At*_^*α*^} satisfying the relation in Eqs. (5) and (7). The average success probability can be written as

12 $${{\mathbb{P}}}_{\mathrm{3,3}}=\frac{1}{9}[\mathop{\sum }\limits_{x\mathrm{=1}}^{3}\,\mathop{\sum }\limits_{y\mathrm{=1}}^{3}\,P(a\ne b|x,y;x=y)+P(a=b|x,y;x\ne y)]$$

writing *P*(*a*, *b*|*x*, *y*) as a moment average $P(a,b|x,y)=\frac{1}{4}\mathrm{[1}+a\langle x\rangle +b\langle y\rangle +ab\langle xy\rangle ]$, it can shown that ${{\mathbb{P}}}_{\mathrm{3,3}}$ is solely dependent on a Bell-like expression *β*_3,3_ is given by

13 $${{\mathbb{P}}}_{\mathrm{3,3}}=\frac{1}{2}[1+\frac{\langle {\beta }_{\mathrm{3,3}}\rangle }{9}]$$

where *β*_3,3_ = (−*A*_1_ + *A*_2_ + *A*_3_) ⊗ *B*_1_ + (*A*_1_ − *A*_2_ + *A*_3_) ⊗ *B*_2_ + (*A*_1_ + *A*_2_ − *A*_3_) ⊗ *B*_3_.

A simple choices of Alice’s observables *A*_1_ = *σ*_*z*_, ${A}_{2}=\frac{\sqrt{3}}{2}{\sigma }_{x}-\frac{1}{2}{\sigma }_{z}$ and ${A}_{2}=\frac{-\sqrt{3}}{2}{\sigma }_{x}-\frac{1}{2}{\sigma }_{z}$ and Bob’s observables *B*_1_ = −*A*_1_, *B*_2_ = −*A*_2_ and *B*_3_ = −*A*_3_ provide the maximum quantum value (*β*_3,3_)_*Q*_^*max*^ = 6. This in turn fixes the maximum average success probability in quantum theory is given by

14 $${({{\mathbb{P}}}_{\mathrm{3,3}})}_{Q}\le \frac{1}{2}(1+\frac{2}{3})\approx 0.833.$$

In two-party, two-outcome Bell scenario, the assumption of preparation non-contextuality is equivalent to locality. In such a case, (*β*_3,3_)_*local*_ ≤ 5 and ${({{\mathbb{P}}}_{\mathrm{3,3}})}_{local}=\mathrm{7/9}\approx 0.777$. This is obtained by assuming that *B*_1_, *B*_2_ and *B*_3_ may take any value in between −1 and +1. But, given the observable choices in quantum theory that maximimizes ${({{\mathbb{P}}}_{\mathrm{3,3}})}_{Q}$ the Bob’s observables satisfy the relation given by Eq. (3) and consequently *B*_1_ + *B*_2_ + *B*_3_ = 0 has to be satisfied. In ontological model Eq. (4) needs to be satisfied which is measurement-noncontextuality assumption. Thus, in an universal non-contextual model (*β*_3,3_)_*unc*_ ≤ 4 and the average success probability is given by

15 $${({{\mathbb{P}}}_{3,3})}_{unc}\le \frac{1}{2}(1+\frac{4}{9})\approx 0.722.$$

Interestingly, the values of $({{\mathbb{P}}}_{\mathrm{3,3}})\in \mathrm{[0.722,}\,\mathrm{0.777]}$ do not reveal the non-classicality in the form of quantum nonlocality but in the form of quantum universal contextuality.

### Generalization for (*n*, *n*) scenario for odd *n*

We generalize the universal quantum contextuality proof for any arbitrary preparation and measurements, i.e., the (*n*, *n*) scenario. Consider the following *n* (odd) number of observables *A*_*n*,1_ = *σ*_*z*_, ${\{{A}_{n,i}\}}_{i=2,\mathrm{...}\frac{n+1}{2}}={\alpha }_{i}{\sigma }_{x}-{\beta }_{i}{\sigma }_{y}-\frac{{\sigma }_{z}}{(n-1)}$ and ${\{{A}_{n,j}\}}_{j=\frac{n+3}{2},\mathrm{...}n}=-\,{\alpha }_{j}{\sigma }_{x}+{\beta }_{j}{\sigma }_{y}-\frac{{\sigma }_{z}}{(n-1)}$ with *α*_*i*_ = −*α*_*j*_, *β*_*i*_ = −*β*_*j*_ and ${\alpha }_{i}^{2}+{\beta }_{i}^{2}+\frac{1}{n-1}=1$. In quantum theory, such choices of observables satisfy ${A}_{n,1}+\mathop{\sum }\limits_{i=2}^{\frac{n+1}{2}}\,{A}_{n,i}+\mathop{\sum }\limits_{j=\frac{n+3}{2}}^{n}\,{A}_{n,j}=0$ and consequently the corresponding projectors satisfy the relation

16 $$\frac{1}{n}({P}_{A\mathrm{,1}}^{+}+\mathop{\sum }\limits_{i\mathrm{=2}}^{\frac{n+1}{2}}\,{P}_{{A}_{n,i}}^{+}+\mathop{\sum }\limits_{j=\frac{n+3}{2}}^{n}\,{P}_{{A}_{n,j}}^{+})=\frac{{\mathbb{I}}}{2}.$$

This relation will provide the non-trivial preparation and measurement non-contextual assumptions along with the trivial assumptions originate from ${\mathbb{I}}={P}_{{A}_{n,t}}^{+}+{P}_{{A}_{n,t}}^{-}$ where *t* = 1, 2 ... *n*. A logical proof of preparation and measurement contextuality can also be demonstrated. Now, in (*n*,*n*) scenario, the average correlation can be written as

17 $${\Delta }_{n}=\frac{1}{2n}\mathop{\sum }\limits_{t=1}^{n}\,\sum _{\alpha \in \{+,-\}}\,p(\alpha |{P}_{{A}_{n,t}}^{\alpha },{P}_{{A}_{n,t}}^{\alpha }).$$

In quantum theory, (Δ_*n*_)_*Q*_ = 1. Following the approach adopted earlier, the average correlation in ontological model is

18 $$\begin{array}{c}{\Delta }_{n}\le \frac{1}{n}\mathop{\sum }\limits_{t\mathrm{=1}}^{n}\,\eta ({P}_{{A}_{n,t}},\lambda )(\frac{1}{2}\sum _{\alpha \in \{+,-\}}\,\mu (\lambda |{\rho }_{{A}_{n,t}}^{\alpha }))\\ \,\,=\,\frac{1}{n}\mathop{\sum }\limits_{t\mathrm{=1}}^{n}\,\eta (\lambda |{P}_{{A}_{n,t}})\nu (\lambda |{\mathbb{I}}/\mathrm{2).}\end{array}$$

According to the mixed state preparation non-contextuality assumption $\nu (\lambda |{\mathbb{I}}/\mathrm{2)}=\frac{1}{2}(\mu (\lambda |{\rho }_{{A}_{n,t}}^{+})+\mu (\lambda |{\rho }_{{A}_{n,t}}^{-}))$ which is independent of *t*. By noting that it is only relevant if *λ* is in the support of $\nu (\lambda |\frac{{\mathbb{I}}}{2})$, we have

19 $${({\Delta }_{n})}_{unc}\le \mathop{\lambda \in \Lambda }\limits^{\max }(\frac{1}{n}\mathop{\sum }\limits_{t=1}^{n}\,\eta (\lambda |{P}_{{A}_{n,t}})).$$

Using equivalent representation of Eq. (16) in a measurement non-contextual ontological model and assigning indeterminstic values of the response function, one has ${({\Delta }_{n})}_{unc}\le 1-\frac{1}{2n}$. Thus, (Δ_*n*_)_*unc*_ < (Δ_*n*_)_*Q*_. However, when *n* is very large (Δ_*n*_)_*unc*_ ≈ (Δ_*n*_)_*Q*_. This is due to the fact that {*η*(*λ*|*P*_*An*,*t*_)} contains only one indeterminstic response function and (2*n* − 1) dsterminstic values out of 2*n* assignments.

Next, we generalize the communication games for (*n*, *n*) scenario where *n* is odd. Let Alice and Bob receive inputs *x* ∈ {1, 2, ... *n*} and *y* ∈ {1, 2, .... *n*} respectively and their respective outputs are *a* ∈ {−1, 1} and *b* ∈ {−1, 1}. The winning rule remains same, i.e., if *x* = *y* then *a* ≠ *b* and if if *x* ≠ *y* then *a* = *b*. The average success probability is given by

$${{\mathbb{P}}}_{n,n}=\frac{1}{{n}^{2}}[\mathop{\sum }\limits_{x,y=1}^{n}\,P(a\ne b|x,y;x=y)+P(a=b|x,y;x\ne y)]$$

which can be cast as

20 $${{\mathbb{P}}}_{n,n}=\frac{1}{2}+\frac{{\beta }_{n}}{2{n}^{2}}$$

where *β*_*n*_ is the Bell expression is given by

21 $${\beta }_{n,n}=\mathop{\sum }\limits_{x,y=1;x\ne y}^{n}\,\langle {A}_{x}{B}_{y}\rangle -\mathop{\sum }\limits_{x,y=1;x=y}^{n}\,\langle {A}_{x}{B}_{y}\rangle .$$

Given the choices of observables satisfy Eq. (16), in quantum theory (*β*_*n*,*n*_)_*Q*_ = 2*n* providing quantum success probability

22 $${({{\mathbb{P}}}_{n,n})}_{Q}=\frac{1}{2}+\frac{1}{n}.$$

For a universal non-contextual model satisfying the equivalent representation of the Eq. 16) in the ontological model, we have (*β*_*n*,*n*_)_*unc*_ = 2*n* − 2 which provides the average success probability in an universal non-contextual model

23 $${({{\mathbb{P}}}_{n,n})}_{unc}=\frac{1}{2}+\frac{1}{n}-\frac{1}{{n}^{2}}.$$

Since ${({{\mathbb{P}}}_{n,n})}_{Q} > {({{\mathbb{P}}}_{n,n})}_{unc}$ for any arbitrary *n*, the universal quantum contextuality is revealed through the communication game.

We make a few comments on the above proofs of universal quantum contextuality. For the above special choices of observables, whenever preparation non-contextuality for mixed state in an ontological model is assumed, one may argue that the preparation contextuality for pure state is automatically installed within the onlogical model. One may then say that inequality (19) for any odd *n* does not provide a true test of universal quantum contextuality. This is due to the fact that assumption of preparation non-contextuality leads us to assume preparation contextuality for pure states and vice versa. However, this feature was not required to be used in the derivation of the inequality (19). In the Appendix, we provide an example of (4, 4) scenario where no logical proof of preparation and measurement contextuality can be demonstrated and no contradiction with quantum theory through the inequality similar to Eq. (19) can be shown. It would then be interesting if the violation of universal non-contextuality can be shown when there is no logical proof possible. We provide such a proof through the communication game in (3, 4) scenario.

### Communication games in (4, 3) and (3, 4) scenarios

Before presenting the communication game in (3, 4) scenario we first demonstrate the communication game in (4, 3) scenario. The game in (4, 3) scenario has close resemblance with the 3 to 1 parity-oblivious random access code. In^13,15^, it was shown that how quantum preparation contextuality powers 3 to 1 random access code.

In (4, 3) scenario, Alice and bob measures four and three dichotomic observables respectively. The winning rule is the following; if *x* + *y* = 5 output requires *a*≠*b* and if *x* + *y* ≠ 5 output requires *a* = *b*. The average success probability can be written as

24 $${{\mathbb{P}}}_{4,3}=\frac{1}{12}[\mathop{\sum }\limits_{x=1}^{4}\,\mathop{\sum }\limits_{y=1}^{3}\,P(a\ne b|x,y;x+y=5)+P(a=b|x,y;x+y\ne 5)]$$

which can be recast as

25 $${{\mathbb{P}}}_{\mathrm{4,3}}=\frac{1}{2}+\frac{\langle {\beta }_{\mathrm{4,3}}\rangle }{24}$$

where *β*_4,3_ = (*A*_1_ + *A*_2_ + *A*_3_ − *A*_4_) ⊗ *B*_1_ + (*A*_1_ + *A*_2_ − *A*_3_ + *A*_4_) ⊗ *B*_2_ + (*A*_1_ − *A*_2_ + *A*_3_ + *A*_4_) ⊗ *B*_3_ is known as Gisin’s elegant Bell expression. The maximization of *β*_4,3_ in turn provides the optimal success probability of the (4, 3) game. In quantum theory, if the projectors with +1 eigenvalues corresponding to Alice’s observables forms a four-outcome SIC-POVM and Bob’s observables are mutually unbiased basis, then ${({\beta }_{4,3})}_{Q}^{opt}=4\sqrt{3}$^31^. Then, ${({{\mathbb{P}}}_{4,3})}_{Q}^{opt}=(1+1/\sqrt{3})/2\approx 0.788$. A model assuming only trivial non-contextuality is in fact a local model. In such a case, (*β*_4,3_)_*local*_ ≤ 6 and (*P*_4,3_)_*local*_ = (1 + 1/2)/2 = 0.75. However, if one assumes additional non-trivial non-contextuality assumption (given in detail in Appendix), a constraint *A*_1_ = *A*_2_ + *A*_3_ + *A*_4_ on Alice observables needs to be satisfied. The operational equivalence in quantum theory dictates to assume such non-contextuality, i.e., functional relation between *A*_*t*_ in the ontological model. Under such constraint of non-contextuality, the preparation-noncontextual bound on Bell expression (*β*_4,3_)_*pnc*_ ≤ 4 and the average success probability is given by ${({{\mathbb{P}}}_{4,3})}_{pnc}=(1+1/3)/2\approx 0.666$. The importance of (4, 3) game example is that the addition of non-trivial non-contextuality assumption along with the trivial ones do not provide the logical contradiction of preparation non-contextuality for pure or mixed state. This thus truly the test of preparation contextuality free from logical inconsistency.

Due to the symmetry, the communication game in (4, 3) scenario can easily be converted to (3, 4) one by swapping the role of Alice and Bob’s observables. In that case it becomes a proof of universal contextuality. Let Alice performs measurement of three mutually unbiased bases. So that, there is no functional relation between Alice three observables and thus no non-trivial preparation non-contextuality assumption can be made. In other words, no logical proof of preparation contextuality can be shown for such choices of observables. For Bob’s choices of observables {*B*_*t*_} (where *t* = 1, 2, 3, 4) along with the trivial conditions $\frac{{\mathbb{I}}}{2}=\frac{1}{2}({P}_{{B}_{t}}^{+}+{P}_{{P}_{t}}^{-})$ the POVM measurements are (the non-trivial conditions)

26 $$\frac{{\mathbb{I}}}{2}=\frac{1}{4}({P}_{{B}_{1}}^{+}+\mathop{\sum }\limits_{t=2}^{4}\,{P}_{{B}_{t}}^{-});\,\frac{{\mathbb{I}}}{2}=\frac{1}{4}({P}_{{B}_{1}}^{-}+\mathop{\sum }\limits_{t=2}^{4}\,{P}_{{B}_{t}}^{+})$$

here, each *P*_*At*_^*α*^ = (1 + *αB*_*t*_)/2 is a rank one projector corresponding to dichotomic observables {*B*_*t*_} having eigenvalues *α* ∈ {1, −1}. To satisfy the relations in Eq. (26) one requires the functional relation *B*_1_ = *B*_2_ + *B*_3_ + *B*_4_ to be satisfied. This is valid even for deterministic and measurement non-contextual values of the response functions. Hence, no logical proof of measurement non-contextuality can be demonstrated irrespective of the nature of the response function.

By keeping the winning rule same, in (3, 4) scenario, the success probability is given by

27 $${{\mathbb{P}}}_{3,4}=\frac{1}{12}[\mathop{\sum }\limits_{x=1}^{3}\,\mathop{\sum }\limits_{y=1}^{4}\,P(a\ne b|x,y;x+y=5)+P(a=b|x,y;x+y\ne 5)]$$

which can be re-written in the following form

28 $${{\mathbb{P}}}_{3,4}=\frac{1}{2}+\frac{\langle {\beta }_{3,4}\rangle }{24}$$

where *β*_3,4_ = *β*_4,3_ and maximum quantum values is $4\sqrt{3}$ for the choices of the observables given in the Appendix. Thus the universal non-contextual bound for *(β*_3,4_)_*unc*_ ≤ 4 providing the success probability ${({{\mathbb{P}}}_{3,4})}_{unc}\le 0.666$ and quantum theory violates this bound. Hence, we have provided a proof to reveal universal quantum contextuality where there is no logical proof of preparation or measurement contextuality exist for the set of choices of the observables in quantum theory. This thus can be considered as a true test of universal quantum contextuality.

## Summary and Discussions

In this report, we discussed the notions noncontextuality in an ontological model of quantum theory which is beyond the traditional notion of KS noncontextuality. Such an ontological model is considered to be universally noncontextual if both the preparation and the measurement noncontextuality assumptions are satisfied in that model. By considering the qubit system, we first generalized a known logical proof of universal quantum contextuality^29^ for any arbitrary odd number of preparation and measurement scenario. We then showed that how such a logical proof can be cast into a universal non-contextual inequality for any odd number of preparation and measurement scenario and demonstrated the quantum violation of them.

In order to demonstrate how universal quantum contextuality powers a communication task, we proposed a two-party communication game where Alice uses *n* (odd) number of dichotomic observables to steer the state of the Bob who also performs the measurement of *n* number of same observables. For any arbitrary *n*, it is shown that the average success probability of winning such game is solely dependent on a suitable Bell expression. For bipartite dichotomic measurements Bell scenario the trivial preparation non-contextuality is equivalent to locality assumption^31,32^. We have shown that the local bound of such a Bell expression gets reduced if non-trivial preparation and measurement non-contextuality conditions i.e., universal non-contextuality is further assumed. Thus, for a given state, even if quantum theory does not violate local realist bound, there is a possibility to reveal non-classicality through the violation of universal non-contextuality.

We have also pointed out the subtleties involved in the logical proof of quantum preparation and measurement contextuality for a suitable set of pure qubit states corresponding to a suitable set of dichotomic observables. If there exists a logical proof of preparation contextuality for mixed state then there will be an inherent interplay between the preparation contextuality for mixed and pure states. In other words, in order to impose the assumption of preparation non-contextuality for a mixed state in an ontological model, the assumption of preparation contextuality for pure states constituting the relevant mixed state requires to be assumed. Such contradiction appears within the framework of concerned ontological model devoid of any operational theory. If one wishes to truly test the universal non-contextuality such inconsistency needs to be avoided. For this, we demonstrated that for a suitable choices of states and observables when is no logical proof of preparation and measurement contextuality can exist in (4,3) scenario, universal quantum contextuality can still be revealed through a suitable communication game. However, for even (*n*,*n*) no logical or statistical proof of universal contextuality can be demonstrated for the qubit system. All the proofs provided in this paper is derived for qubit system and thus experimentally testable using existing technology. The precise operational equivalence has to be ensured in the real experiments following a recently developed approach^29,32^.

## Supplementary information

Supplementary information
