# Author Correction: Revealing universal quantum contextuality through communication games

**DOI:** 10.1038/s41598-020-64337-1

**Published:** 2020-04-30

**Authors:** A. K. Pan

**Affiliations:** National Institute of Technology Patna, Ashok Rajhpath, Patna, 800005 India

Correction to: *Scientific Reports* 10.1038/s41598-019-53701-5, published online 26 November 2019

The Acknowledgements section in this Article is incorrect.

“Author gratefully acknowledges the discussion with Prof. G. Kar (ISI, Kolkata) and the support from the project DST/ICPS/QuST/Theme-12019/4.”

should read:

“Author gratefully acknowledges the discussion with Prof. G. Kar (ISI, Kolkata) and the support from the Ramanujan Fellowship Research Grant (No. SB/S2/RJN-083/2014)”.

