# Supplementary material for: Revealing universal quantum contextuality through communication games
Source: Sci Rep. 2019 Nov 26;9:17631. doi: 10.1038/s41598-019-53701-5 (PMC6879595; doi:10.1038/s41598-019-53701-5)
Supplement: Supplementary file 1 — Supplementary information [file 41598_2019_53701_MOESM1_ESM.pdf]

Revealing universal quantum contextuality through communication games

A. K. Pan

Appendix A: THE FOUR-PREPARATION AND FOUR-MEASUREMENT $(4, 4)$ SCENARIO

:

We show that in four-preparation and four-measurement scenario (the $(4, 4)$ scenario) no logical contradiction of preparation and measurement non-contextuality can also be demonstrated. We further show that in such case no contradiction with quantum theory through the inequality similar to equation (19) can be demonstrated. We then propose a communication game similar to $(4, 4)$ scenario to show that there is no contradiction as well.

Let four preparation procedures $\{P_t\} \in \mathcal{P}$ where $t = 1, 2, 3, 4$ realize the observables A_t and produce eight pure qubit states $\{\rho^{\alpha}_{A_t}\}$ satisfying following four relations

$$\frac{\mathbb{I}}{2} = \frac{1}{2}(\rho^{+}_{A_t} + \rho^{-}_{A_t}) \tag{A1}$$

In an ontological model, the λ distribution can be written as

$$\mu_{P_t}(\lambda|\frac{\mathbb{I}}{2}) = \frac{1}{2}\left(\mu_{P_t}(\lambda|\rho^{+}_{A_t}) + \mu_{P_t}(\lambda|\rho^{-}_{A_t})\right) \tag{A2}$$

which are the trivial preparation non-contextuallity condition.

If eight qubit projectors $\{\rho^{\alpha}_{A_t}\}$ are obtained from Alice's measurement of four observables are $A_1 = (\sigma_x+\sigma_y+\sigma_z)/\sqrt{3}$, $A_2 = (\sigma_x + \sigma_y - \sigma_z)/\sqrt{3}$, $A_3 = (\sigma_x - \sigma_y + \sigma_z)/\sqrt{3}$ and $A_4 = (-\sigma_x + \sigma_y + \sigma_z)/\sqrt{3}$. Then the maximally mixed state $\frac{\mathbb{I}}{2}$ can also be prepared by two more preparation procedures P_5 and P_6 are of the following form

$$\frac{\mathbb{I}}{2} = \frac{1}{4}\Big(\rho^{+}_{A_1} + \sum_{t=2}^{3}\rho^{-}_{A_t}\Big); \qquad \frac{\mathbb{I}}{2} = \frac{1}{4}\Big(\rho^{-}_{A_1} + \sum_{t=2}^{3}\rho^{+}_{A_t}\Big) \tag{A3}$$

If ontological model is preparation non-contextual for mixed states, then $\mu_{P_1}(\lambda|\frac{\mathbb{I}}{2}) = \mu_{P_2}(\lambda|\frac{\mathbb{I}}{2}) = \mu_{P_3}(\lambda|\frac{\mathbb{I}}{2}) = \mu_{P_4}(\lambda|\frac{\mathbb{I}}{2}) = \mu_{P_5}(\lambda|\frac{\mathbb{I}}{2}) = \mu_{P_6}(\lambda|\frac{\mathbb{I}}{2}) = \nu(\lambda|\frac{\mathbb{I}}{2})$. One can then write

$$\nu(\lambda) = \frac{1}{2}\Big(\mu_{P_t}(\lambda|\rho^{+}_{A_t}) + \mu_{P_t}(\lambda|\rho^{-}_{A_t})\Big) \tag{A4a}$$

$$= \frac{1}{4}\left(\mu_{P_5}\left(\lambda|\rho^{+}_{A_1}\right) + \sum_{t=2}^{4}\mu_{P_5}\left(\lambda|\rho^{-}_{A_t}\right)\right) \tag{A4b}$$

$$= \frac{1}{4}\left(\mu_{P_5}\left(\lambda|\rho^{-}_{A_1}\right) + \sum_{t=2}^{4}\mu_{P_5}\left(\lambda|\rho^{+}_{A_t}\right)\right) \tag{A4c}$$

Let us now consider the values of those epistemic states for a fixed λ. Since $\rho_{A_t^+}$ is orthogonal to $\rho_{A_t^-}$, then there is no common λ in the support of both $\mu(\lambda|\rho_{A_t^+})$ and $\mu(\lambda|\rho_{A_t^+})$, so that $\mu(\lambda|\rho_{A_t^+})\mu(\lambda|\rho_{A_t^-}) = 0$.

If for a given λ, we have $\mu_{A_t}(\lambda|\rho_{A_1^+})$, $\mu(\lambda|\rho_{A_2^-})$, $\mu(\lambda|\rho_{A_3^-})$ and $\mu(\lambda|\rho_{A_4^-})$ are zero, then from equation(A4b) we have $\nu(\lambda)$ is zero which contradicts with all other conditions. But, if one assumes the λ is in the support of $\mu(\lambda|\rho_{A_1^+})$, $\mu(\lambda|\rho_{A_2^-})$, $\mu(\lambda|\rho_{A_3^+})$ and $\mu(\lambda|\rho_{A_4^+})$, then equations (A4a- A4c)) can be written as

$$\nu(\lambda) = \frac{1}{2}\Big(\mu_{P_3}(\lambda|\rho^{+}_{A_3})\Big) \tag{A5a}$$

$$= \frac{1}{2}\Big(\mu_{P_4}(\lambda|\rho^{+}_{A_4})\Big) \tag{A5b}$$

$$= \frac{1}{4}\Big(\mu_{P_6}(\lambda|\rho^{+}_{A_3}) + \mu_{P_6}(\lambda|\rho^{+}_{A_4})\Big) \tag{A5c}$$

It can be easily checked from equations(A5a-A5c) that they are consistent and no logical proof of preparation contextuality for the above scenario.

From equations (29) and (31) in an ontological model, one can write

$$1 = \xi_{M_t}(+|P^{+}_{A_t}) + \xi_{M_t}(+|P^{-}_{A_t}) \tag{A6a}$$

$$\frac{1}{2} = \frac{1}{4}\left(\xi_{M_5}(+|P^{+}_{B_t}) + \sum_{t=2}^{4}\xi_{M_5}(+|P^{-}_{t})\right); \quad \frac{1}{4}\left(\xi_{M_6}(+|P^{-}_{B_t}) + \sum_{t=2}^{4}\xi_{M_6}(+|P^{+}_{t})\right) \tag{A6b}$$

It can be easily shown that all the response functions may take determinstic value and non-contextual.

Next, the average correlation for $(4,4)$ scenario in quantum theory can be written as

$$\Delta_{4,4} = \frac{1}{8}\sum_{t=1}^{4}\sum_{\alpha\in\{+,-\}} p(\alpha|\rho^{\alpha}_{t}, P^{\alpha}_{A_t}) \tag{A7}$$

Note that in quantum theory, $(\Delta_{4,4})_Q = 1$. In an ontological model as

$$\Delta_{4,4} = \frac{1}{8}\sum_{t=1}^{4}\sum_{\alpha\in\{+,-\}}\sum_{\lambda\in\Lambda}\xi(\alpha|P^{\alpha}_{A_t},\lambda)\mu(\lambda|\rho^{\alpha}_{A_t}) \tag{A8}$$

In order to get perfect average correlation, the each term in the equation (A8) needs to be perfectly correlated, implying that every response function $\xi(\alpha|P^{\alpha}_{A_t},\lambda)$ should produce deterministic outcome. Now, applying trivial preparation non-contextuality as in Ref. [32], we can write

$$(\Delta_{4,4})_{unc} \leq \overset{max}{\lambda \in \Lambda}\left(\frac{1}{4}\sum_{t=1}^{4}\eta(P_{A_t},\lambda)\right) \tag{A9}$$

The quantity $\eta(P_{A_t},\lambda)$ can be maximized with the condition given in equations (29) and (7). It is simple to show that $\Big(\eta(P_{A_1},\lambda),\eta(P_{A_2},\lambda),\eta(P_{A_3},\lambda),\eta(P_{A_4},\lambda)\Big) = \Big(1,1,1,1\Big)$. We then have , $(\Delta_{4,4})_{unc} = (\Delta_{4,4})_Q = 1$. Thus, in this case universal non-contextual model can reproduce the perfect predictability of quantum theory. We can then argue that for even (n,n) scenario, all the response functions can take $\eta(P_{A_t},\lambda)$ can take deterministic value 1 and there is no contradiction between quantum theory and universal noncontextuality can be shown.

A communication game in $(4,4)$ scenario:

Let Alice and Bob are two parties having input $x \in \{1,2,3,4\}$ and $y \in \{1,2,3,4\}$ respectively and outputs are $a \in \{-1,1\}$ and $b \in \{-1,1\}$. The wining rule is the following. If $x = y$ the outputs satisfies $a \neq b$ and if $x \neq y$ the outputs satisfies $a = b$. Let the input $x \in \{0,1,2,3\}$ corresponds to A_1, A_2, A_3, A_4 and $y \in \{0,1,2\}$ corresponds to B_1, B_2, B_3 with $A_i = B_i$. The average success probability can be written as

$$\mathbb{P} = \frac{1}{16}\Big[\sum_{x,y=1}^{4}\Big[P(a\neq b|x,y;x=y) + P(a=b|x,y;x\neq y)\Big]\Big] \tag{A10}$$

which can then be cast as

$$\mathbb{P}_{4,4} = \frac{1}{2} + \frac{\langle\beta_4\rangle}{32} \tag{A11}$$

where

$$\begin{aligned}\beta_{4,4} = {} & A_1\otimes(-B_1+B_2+B_3+B_4) + A_2\otimes(B_1-B_2+B_3+B_4) \\ & + A_3\otimes(B_1+B_2-B_3+B_4) + A_4\otimes(B_1+B_2+B_3-B_4)\end{aligned} \tag{A12}$$

It can be shown that $(\beta_{4,4})_{unc} = (\beta_{4,4})_Q \leq 8$. Universal quantum contextuality cannot be demonstrated through the $(4,4)$ game considered here. This argument should hold true in (n,n) scenario for even n and qubit quantum system.
